# Shortened Relative Leukocyte Telomere Length Is Associated With Polycystic Ovary Syndrome and Metabolic Traits

**DOI:** 10.1002/edm2.70030

**Published:** 2025-02-18

**Authors:** Raymond N.C. Chan, Chuiguo Huang, Noel Y. H. Ng, Henry C. H. Tam, Claudia H. T. Tam, Feifei Cheng, Kwun Kiu Wong, Mai Shi, Alex C. W. Ng, Atta Y. T. Tsang, Chi Chiu Wang, Lai Ping Cheung, Wing Hung Tam, Mugdha V. Joglekar, Anandwardhan A. Hardikar, Alicia J. Jenkins, Juliana C. N. Chan, Cadmon K. P. Lim, Ronald C. W. Ma

**Affiliations:** ^1^ Department of Medicine and Therapeutics The Chinese University of Hong Kong, Prince of Wales Hospital Hongkong China; ^2^ Department of Obstetrics and Gynaecology The Chinese University of Hong Kong, Prince of Wales Hospital Hongkong China; ^3^ Li Ka Shing Institute of Health Sciences The Chinese University of Hong Kong Hongkong China; ^4^ Diabetes and Islet Biology Group, School of Medicine Western Sydney University Sydney New South Wales Australia; ^5^ Baker Heart and Diabetes Institute Melbourne Victoria Australia; ^6^ NHMRC Clinical Trial Centre, Faculty of Medicine and Health University of Sydney Sydney New South Wales Australia; ^7^ Hong Kong Institute of Diabetes and Obesity, The Chinese University of Hong Kong Hongkong China; ^8^ Chinese University of Hong Kong‐Shanghai Jiao Tong University Joint Research Centre in Diabetes Genomics and Precision Medicine Hongkong China

**Keywords:** dysglycemia, dyslipidemia, leukocyte telomere length, polycystic ovary syndrome

## Abstract

**Background:**

Polycystic ovary syndrome (PCOS) is one of the commonest gyneco‐endocrine disorders amongst women of reproductive age. Whether PCOS and cardiometabolic traits in PCOS patients are associated with shortened telomere length (TL) or relative leukocyte telomere length (rLTL) remains unclear.

**Methods:**

214 women with PCOS and 214 age‐matched women were recruited. rLTL was measured with an updated quantitative real‐time PCR protocol and reported as ΔΔCt between telomere and a single‐copy gene encoding β‐globin relative to a normalisation control. A two‐way Mendelian randomization analysis using the UK Biobank Resource was performed to assess the causal relationship between rLTL and PCOS.

**Results:**

Women with PCOS had significantly shortened rLTL (PCOS: 0.5 ± 0.7; control: 0.8 ± 0.6; *p* < 0.001). Longer rLTL was associated with a lower risk of PCOS after adjusting for age, history of smoking and other cardiometabolic traits (OR: 0.503; 95% CI: 0.342–0.730; *p* < 0.001). Longer rLTL was associated with reduced risk of dyslidpidemia (OR: 0.563; 95% CI: 0.450–0.968; *p* = 0.042) in PCOS patients. PCOS subjects with rLTL shorter than mean of the rLTL of control subjects had an elevated risk of dysglycemia (OR: 2.09; 95% CI: 1.04–4.29; *p* = 0.040). No causal relationships were found between rLTL and PCOS in the Mendelian randomization study.

**Conclusions:**

Women with PCOS have significantly reduced rLTL and shorter LTL may be associated with cardiometabolic risk factors in PCOS subjects. There are no causal relationship between genetically determined PCOS and TL or vice versa.

## Graphical abstract

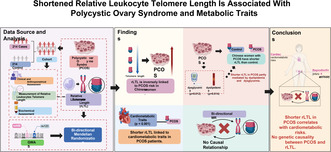

## 1 Introduction

Polycystic ovary syndrome (PCOS) is one of the commonest endocrine disorders amongst women of reproductive age, and is characterised by clinical/biochemical hyperandrogenism, chronic oligo/amenorrhea with or without polycystic ovary morphology [1, 2]. It is associated with elevated risks of various metabolic comorbidities including obesity, insulin resistance, type 2 diabetes mellitus (T2DM), hyperlipidemia and non‐alcohol fatty liver disease, regardless of age and BMI [3, 4, 5, 6, 7]. Current estimates indicate a global prevalence of 5.5%–19.9% and can be up to 70%–80% amongst women with oligomenorrhea, constituting a significant public health burden [8, 9]. Although the epidemiological links between PCOS and these metabolic conditions are relatively well‐established, the exact pathophysiological mechanisms remain unclear and are likely to involve interaction between disturbance of hormonal balance, alteration in cellular metabolism, systemic inflammation and oxidative stress [10].

Telomeres are specialised nucleoprotein structures at the ends of each DNA strand in the chromosomes of all eukaryotic cells. It is composed of tandem repeats of 5′‐TTAGGG‐3′ sequences bound by shelterin to act as a protective structure of the DNA during cell division. Due to the abundance of guanine, telomeres are intrinsically sensitive to oxidative damage and are proposed to be a potential marker of cellular ageing as well as oxidative stress in humans [11]. Previous studies have reported shortened telomere length in numerous age‐related conditions including T2DM, chronic kidney disease, neurocognitive disorders and malignancies [12, 13, 14, 15, 16]. The association between gynaecological conditions, such as infertility, has also been implicated [17, 18]. In PCOS, whether or not telomere length reflects and could serve as a potential marker for certain pathophysiological changes including cardiometabolic derangement, remains a question.

Several groups have evaluated the relationship between telomere length and PCOS, but the results have been inconsistent. A case–control study reported significantly shorter leukocytes telomere length (LTL) in 698 subjects with PCOS, after adjusting for age and their results were echoed by another group in Brazil in 2020 [19, 20]. However, some earlier studies have reported no differences in telomere length between women with PCOS and control [20, 21]. A recent systematic review and meta‐analysis of six studies likewise concluded no significant difference in telomere length in women with or without PCOS [22]. Some of the discrepancies may be related to the variation in LTL measurements depending on the methods used, and these apparently contradicting results warrant further investigation into the association between telomere length and PCOS using more accurate assays [12]. Hence, the present study aims to compare the relative leukocyte telomere length (rLTL) in PCOS and control subjects, using a refined and medium throughput assay with greater accuracy [23]. In addition, we evaluated the relationship between rLTL and various clinical or biochemical characteristics in PCOS subjects, as well as explored causal links between PCOS and LTL.

## 2 Methods

### 2.1 PCOS Patients and Control Subjects

A total of 214 women with PCOS were recruited from the endocrine, combined gynae‐endocrine clinics and PCOS registry in the Prince of Wales Hospital. Baseline date was defined as the date of PCOS diagnosis by relevant endocrinologists or gynaecologists between September 1999 and April 2007. Each patient has been further confirmed to have PCOS based on the 2003 Rotterdam criteria before enrolling to the present study. These patients were then followed‐up and re‐assessed between January 2016 and December 2017 [24]. Using the existing cohort, several studies have been conducted on the progression of cardiometabolic risk in women with PCOS [24, 25, 26].

Another 214 age‐matched (± 3 years to the age of patients at follow‐up visits) control subjects were recruited from the Hyperglycemia and Adverse Pregnancy Outcome (HAPO) study, who had all previously conceived without assisted reproduction technology, and attended a follow‐up visit between 2009 and 2013, approximately 7 years after delivery [25, 27]. These subjects had been seen by obstetricians during pregnancy and never had a diagnosis of PCOS. They had normal glucose tolerance during pregnancy as screened by standard 75‐g oral glucose tolerance tests (OGTT). Subjects with hypothyroidism, prolactinoma, nonclassical adrenal hyperplasia and Cushing's syndrome were excluded. This study was approved by the Joint Chinese University of Hong Kong‐New Territories East Cluster Clinical Research Ethics Committee. Written informed consent was obtained from all subjects in the study.

### 2.2 Clinical, Anthropometrical and Biochemical Assessment

Personal history, medical history and drug history were documented with a standardised questionnaire. Metabolic risk factors including tobacco use and family history of diabetes were assessed. Oligo/amenorrhea was defined by < 10 menstrual periods in the year before the follow‐up. Clinical hyperandrogenism was evaluated by the degree of hirsutism, the frequency of hair removal and the methods used. We defined hirsutism by the modified Ferriman–Gallwey score with a cut‐off point of ≥ 3, as appropriate for East Asian subjects [1, 28]. Body weight, body height, waist circumference and hip circumference were measured. Systolic and diastolic blood pressure were obtained by averaging two readings by a standard sphygmomanometer on the right arm after at least 5 min of rest.

Overnight‐fasting blood samples were collected in all subjects at the follow‐up visits, as this study aimed to explore the association between rLTL and cardiometabolic risk factors, which usually manifest with increasing age. Fasting blood glucose (FBG), fasting insulin, total cholesterol (TC), triglyceride (TG), high‐density lipoprotein (HDL) and low‐density lipoprotein (LDL) were measured. A 75‐g OGTT was performed in all subjects, unless there is a known diagnosis of DM, or if the subjects had undergone a recent OGTT prior to clinical assessment. In subjects with PCOS, serum follicle stimulating hormone (FSH), luteinizing hormone (LH), progesterone, estradiol (E_2_), testosterone, androstenedione and sex hormone‐binding globulin (SHBG) were measured.

Plasma glucose, TC, TG, HDL and LDL were evaluated by appropriate enzymatic methods on the Cobas c702 Clinical Chemistry Analyser (Roche Diagnostics Corp.). Insulin and SHBG were measured by Immunilite XPi2000 Analyser (Siemens Healthcare Diagnostics Inc.). Electro‐chemiluminescent immunoassays on the Cobas c601 Immunoassay Analyser (Roche Diagnostics Corp.) were employed to measure LH, FSH and E_2_. Liquid chromatography tandem mass spectrometry (LC–MS/MS) was used for the measurement of serum total testosterone and androstenedione [24]. Standardised reagent kits were supplied by the manufacturers and the analytical performance was within the specification of the analysers. The laboratory responsible for these tests is accredited by the National Association of Testing Authority, Australia (NATA) and the Royal College of Pathologists of Australasia against ISO15189:2012 for medical testing.

### 2.3 Outcomes Measures

Subjects with a body mass index ≥ 23 kg/m^2^ or a waist circumference ≥ 80 cm were classified as being overweight or having central obesity, respectively. Dysglycemia was defined by impaired fasting glucose (IFG), impaired glucose tolerance (IGT) or DM by the 2009 American Diabetes Association diagnostic criteria [29]. IFG was defined as FBG between 5.6 mmol/L and 6.9 mmol/L while IGT was defined as 2‐h plasma glucose between 7.8 mmol/L and 11.0 mmol/L. DM was defined by FBG ≥ 7 mmol/L, 2‐h plasma glucose ≥ 11.1 mmol/L or according to patient history in the standard questionaire. Hypertension was defined by the report from the Panel Members in the Eighth Joint National Committee (JNC8), or according to the questionaire. Subjects with TC ≥ 5.2 mmol/L, TG ≥ 1.7 mmol/L, HDL‐C < 1.3 mmol/L, LDL‐C ≥ 3.4 mmol/L, or treatment with lipid regulating drugs were considered to have dyslipidemia. Free androgen index (FAI) was calculated by 100× (total testosterone (nmol/L))/SHBG (nmol/L). Insulin resistance was assessed by the homeostatic model assessment of insulin resistance (HOMA IR), that is, fasting glucose (mmol/L) × fasting insulin (mIU/L)/22.5.

### 2.4 Measurement of Relative Leukocytes Telomere Length and Estimated Absolute Leukocytes Telomere Length

Whole blood samples of all subjects were stored at −80°C until retrieval for DNA extraction. DNA was extracted by the phenol‐chloroform method. We measured rLTL as per an updated quantitative real‐time PCR protocol and reported it as ΔΔCt between telomere and a single‐copy gene encoding β‐globin (HBG) relative to a normalisation control [23]. With this protocol, intra‐assay CV < 2% and inter‐assay CV < 4% were achieved, alongside high reproducibility from three users in two different laboratories [23]. Quality control was achieved by inclusion of a no‐template control (NTC) and a reference human DNA sample (QC), which allowed normalisation of plate‐to‐plate variability, as well as the calculation of ΔΔCt. Currently, there is no consensus on the most appropriate normalisation method for rLTL measurement and calculation based on either NTC or QC was generally considered acceptable. In the present study, we used QC for normalisation. For PCOS subjects, we utilised the DNA sample during the follow‐up visit for measurement of LTL, as this was the timepoint at which subjects were matched. The CV of each sample was < 2.5%. The overall intra‐plate CVs were 1.06% and 0.64% for telomere and HBG respectively, while the inter‐plate CVs were 1.74% and 0.84%.

We also estimated absolute LTL from whole‐genome‐sequencing (WGS) data for whole blood samples using Telseq software [30, 31]. Telseq defines the reads which contained seven or more TTAGGG repeats as telomeres, and thereby calculates the relative proportion of telomeric reads amongst all sequenced reads and transforms this value into absolute telomere length. From our previous analyses, one unit of rLTL measured using our assay was equivalent to 4.999 kilobases of absolute LTL estimated from WGS [31].

### 2.5 Data Sources and SNP Selection for Mendelian Randomization Analysis

Full‐genetic instrument variables (IVs) for rTL were obtained from a GWAS study including 472,174 well‐characterised participants of European descent using the UK Biobank Resource [15] and the relevant summary statistics for PCOS were obtained from the Day et al. study, which is a large GWAS meta‐analysis with 4138 cases and 20,129 controls collated from six European cohorts [32]. The rationale for performing the MR analysis based on Europoean populations is due to the much larger sample size available for the GWAS and hence stronger instrumental variables available in European populations as opposed to East Asian populations, and the expectation that causal relationships established in one population should not be ethnicity‐specific, but should be applicable for other populations.

We employed a conventional threshold of genome‐wide significance (*p* < 5 × 10^−8^) to select the candidates of genetic instruments for the exposure trait. We further removed correlated SNPs by setting a linkage disequilibrium threshold of *r* ^2^ < 0.001. We retained the independent SNPs with the lowest *p*‐values within a 10,000‐kb window. These SNPs were then harmonised with the outcome GWAS summary statistics, where SNPs were discarded if they were either palindromic or not available in the outcome GWAS. The remaining SNPs were used as the final instrumental variables (IVs) for exposure. To validate the instrument strength assumption for MR analysis, we estimated the proportion of variance in the exposure explained by genetic variants (*R* ^2^) using the formula:

$$R^{2}\approx \sum_{i}^{K}\frac{\beta_{i}^{2}}{\beta_{i}^{2}+N{\left(\mathrm{se}\left(\beta_{i}\right)\right)}^{2}}$$

where *β*_i_ is the effect size of genetic instrument variant *i*, *N* is the effective sample size, se(*β*_i_) is the standard error of effect size for the genetic variant *i*, and *K* is the number of independent genetic variants [33]. We then calculated the *F*‐statistic by *F* = ((*N*−K−1)*R* ^2^)/(K(1−*R* ^2^)) and compared it with the empirical threshold 10 to evaluate the strength of these genetic instruments.

The current MR analyses were performed using published GWAS datasets. No specific ethical approval and written informed consent of participants is required for the MR analyses.

### 2.6 Statistical Analysis

rLTL beyond two standard deviations from the mean of either group were excluded from analysis, alongside their matched samples, which included four cases and seven controls together with their matched samples. The distribution of variables was assessed by skewness and kurtosis test. Demographic information was presented in mean ± SD or median (interquartile range) for continuous data, and frequency (percentage) for categorical data. Qualitative and quantitative differences between subjects with PCOS and control subjects were analysed by chi‐square test for categorical variable, and unpaired *t*‐test or Mann–Whitney test for continuous variable, whichever is appropriate. Simple and multivariable logistic regression were applied to examine the association of rLTL/estimated absolute LTL and PCOS. Mediation analysis was performed to evaluate the potential causal relationship between PCOS stuatus, cardiometabolic risk factors and rLTL. Subgroup analyses were performed in subjects with PCOS to evaluate the relationship between rLTL and their clinical/biochemical characteristics. Linear and logistic regression were used for continuous and dichotomous variables respectively. Multivariable analyses were performed to examine the independent association between rLTL and these parameters. We also defined shortened rLTL as rLTL < mean rLTL in control subjects to calculate the unadjusted and adjusted odds ratios between risk of dysglycemia, hypertension, central obesity and shortened rLTL by logistic regression. The MR analyses were undertaken using the R packages TwoSampleMR. The inverse variance‐weighted (IVW) method was used as the determinant of the causal effects of exposure on outcome. We also performed maximum likelihood, weighted median, weighted mode and MR‐Egger methods for validating the primary findings from IVW. Other sensitivity tests including the heterogeneity test (Cochrane's *Q* test), pleiotropy test (MR‐Egger intercept test), and leave‐one‐out test were also performed [33]. Statistical significance was taken as *p* < 0.05. All statistical analyses were two‐tailed and performed using R version 4.0.2.

## 3 Results

### 3.1 Baseline and Follow‐Up Characteristics of Women With PCOS and Controls

A total of 203 women with PCOS and 203 control subjects were included in the statistical analyses. At the time of previous assessment, 85 (58.2%), 159 (97.0%) and 138 (92.6%) of the PCOS subjects had clinical/biochemical hyperandrogenism, oligo/amenorrhea and polycystic morphology respectively, whilst 46 (22.7%), 75 (36.9%) and 22 (10.8%) of them had dysglycemia, dyslipidemia and hypertension.

Compared with the controls, women with PCOS at the follow‐up visits were older and had greater body weight, BMI, waist circumference, waist‐hip ratio (WHR), SBP, DBP, TG, FBG, insulin, blood glucose 2‐h post‐OGTT and HOMA‐IR (all *p* < 0.05). They also had lower HDL, HOMA‐B and shorter rLTL. More of them were overweight/obese and more had DM, dysglycemia, hyperlipidemia, hypertension, central obesity and family history of DM (all *p* < 0.05). It is estimated that absolute LTL amongst PCOS subjects was 13.1 ± 4.5 kilobase pair, compared to 15.0 ± 4 kilobase pair amongst controls.

Androgen and other sex hormones were measured only in the PCOS subjects and the data were presented in Table 1. Meanwhile, control subjects had no clinical features of PCOS, although the androgen level had not been measured. Control women have all previously conceived without receiving any assisted reproduction technology. The baseline and follow‐up characteristics of women with PCOS and controls were summarised in Table 1.

**TABLE 1:** Baseline and follow‐up characteristics of polycystic ovary syndrome (PCOS) patients and controls.

	PCOS patients at baseline, (*n* = 203)	PCOS patients at follow‐up, (*n* = 203)	Controls, (*n* = 203)	*p* ^a^
Baseline demographics				
Age (years)	30.8 ± 6.5	40.7 ± 6.3	39.3 ± 4.9	0.008
Height (m)	157.7 ± 5.2	157.9 ± 5.6	157.8 ± 5.5	0.912
Weight (kg)	65.1 ± 15.2	66.3 ± 14.7	58.1 ± 8.9	< 0.001
Body mass index (kg/m^2^)	26.1 ± 5.7	26.5 ± 5.4	23.3 ± 3.4	< 0.001
Waist circumference (cm)	83.1 ± 12.3	82.9 ± 13.4	78.0 ± 8.9	< 0.001
Hip circumference (cm)	99.0 (14.0)	96.0 (11.8)	96.0 (8.6)	0.539
Waist‐hip ratio	0.8 (0.1)	0.8 (0.1)	0.8 (0.1)	< 0.001
Systolic blood pressure (mm Hg)	117 ± 16.2	119.7 ± 14.3	111.6 ± 11.7	< 0.001
Diastolic blood pressure (mm Hg)	71.8 ± 10.5	78.6 ± 12.7	71.8 ± 9.7	< 0.001
Smoking status				
Current smoker	11 (5.4%)	19 (9.4%)	20 (9.9%)	0.310
Ex‐smoker	8 (3.9%)	8 (3.9%)	15 (7.4%)	
Family history of DM	N/A	108 (53.2%)	57 (28.1%)	< 0.001
Clinical features of PCOS patients				
Biochemical/clinical hyperandrogenism	85 (58.2%)	58 (28.6%)	N/A	N/A
Oligo/amenorrhea	159 (97.0%)	96 (47.3%)	N/A	N/A
Polycystic ovary morphology	138 (92.6%)	N/A	N/A	N/A
Co‐morbidities				
Overweight/obesity	88 (43.3%)	147 (72.4%)	96 (47.3%)	< 0.001
Central obesity	73 (36.0%)	113 (55.7%)	81 (40.0%)	0.002
Dysglycemia	46 (22.7%)	84 (41.4%)	44 (21.7%)	< 0.001
DM	13 (6.4%)	34 (16.7%)	2 (1.0%)	< 0.001
Hypertension	22 (10.8%)	68 (33.5%)	3 (1.5%)	< 0.001
Hyperlipidemia	75 (36.9%)	130 (64.0%)	6 (3.0%)	< 0.001
Medications				
Anti‐hypertensive	3 (1.5%)	43 (21.2%)	1 (0.5%)	< 0.001
Lipid‐lowering drugs	1 (0.5%)	28 (13.8%)	1 (0.5%)	< 0.001
Metabolic profile				
Triglyceride (mmol/L)	1.2 (1.1)	1.1 (0.9)	0.8 (0.6)	< 0.001
Total cholesterol (mmol/L)	4.8 (1.2)	4.9 (0.9)	4.9 (0.9)	0.828
High density lipoprotein (mmol/L)	1.5 (0.5)	1.4 (0.5)	1.6 (0.5)	0.008
Low density lipoprotein (mmol/L)	2.5 (0.9)	2.8 (0.8)	2.8 (0.8)	0.473
Fasting blood glucose (mmol/L)	5.0 (0.7)	4.9 (0.9)	4.7 (0.5)	< 0.001
2‐h glucose post‐OGTT (mmol/L)	6.7 (2.6)	6.7 (2.8)	5.6 (1.9)	< 0.001
Insulin (pmol/L)	72.0 (93.5)	53.9 (51.8)	19.0 (20.9)	< 0.001
HOMA‐IR	2.98 (4.01)	2.0 (2.4)	0.7 (0.8)	< 0.001
HOMA‐B	142.6 (151)	119.3 (94.5)	54.2 (54.0)	< 0.001
Sex hormone				
Luteinizing hormone (IU/l)	7.6 (7.6)	6.5 (5.6)	N/A	N/A
Follicle stimulating hormone (IU/l)	5.85 (2.0)	6.7 (4.3)	N/A	N/A
Estradiol (pmol/L)	135.5 (79.3)	138.5 (218)	N/A	N/A
Testosterone (nmol/L)	1.40 (0.8)	0.8 (0.6)	N/A	N/A
Androstenedione (nmol/L)	N/A	3.7 (2.7)	N/A	N/A
Free androgen index	4.2 (4.7)	3.2 (3.5)	N/A	N/A
Sex hormone binding globulin	33.3 (29.1)	37.2 (30.5)	N/A	N/A
Leukocyte telomere length				
rLTL	N/A	0.5 ± 0.7	0.8 ± 0.6	< 0.001
Estimated absolute LTL (kilobase pair)	N/A	13.1 ± 4.5	15.0 ± 4.0	< 0.001

^a^ *p*‐value for differences between patients with PCOS during follow‐up and control.

### 3.2 Shorter rLTL Is Associated With Increased Odds of PCOS

We performed simple and multiple logistic regression to assess the association between rLTL and PCOS status. The 6 models were summarised in Table 2. In the univariable analysis, rLTL was negatively associated with PCOS (OR: 0.482; 95% CI: 0.342–0.668; *p* < 0.001). To adjust for the effects of age and other cardiometabolic traits on rLTL, five multivariable models were constructed. The association remained significant after serial adjustment for age, waist circumference, smoking, HOMA‐IR and LDL (OR: 0.503; 95% CI: 0.342–0.730; *p* < 0.001) and each unit increase in rLTL was associated with a roughly 49.7% reduction in the risk of PCOS. Sensitivity analyses were performed by adjusting for BMI instead of waist circumference, as well as fasting glucose instead of HOMA‐IR, which generated similar results (Table S8). Based on the absolute LTL data extrapolated from WGS, 1 kilobase increase in absolute LTL was associated with a 10% reduction in risk of PCOS (OR: 0.900; 95% CI: 0.857–0.944, *p* < 0.001), which remained significant after adjustment (OR: 0.906; 95% CI: 0.857–0.956; *p* < 0.001) (Table S7).

**TABLE 2:** Association of PCOS status and rLTL at follow‐up using simple and multivariable logistic regression.

	Odds ratio	95% CI	*p*
Model 1	0.482	0.342–0.668	< 0.001
Model 2	0.505	0.358–0.703	< 0.001
Model 3	0.515	0.362–0.733	< 0.001
Model 4	0.512	0.359–0.719	< 0.001
Model 5	0.530	0.363–0.762	< 0.001
Model 6	0.503	0.342–0.730	< 0.001

*Note:* Model 1: Unadjusted odds ratio. Model 2: Adjusted for age. Model 3: Adjusted for age and waist circumference. Model 4: Adjusted for age, waist circumference, and smoking. Model 5: Adjusted for age, waist circumference, smoking and HOMA IR. Model 6: Adjusted for age, waist circumference, smoking, HOMA IR and LDL.

**FIGURE 1:**
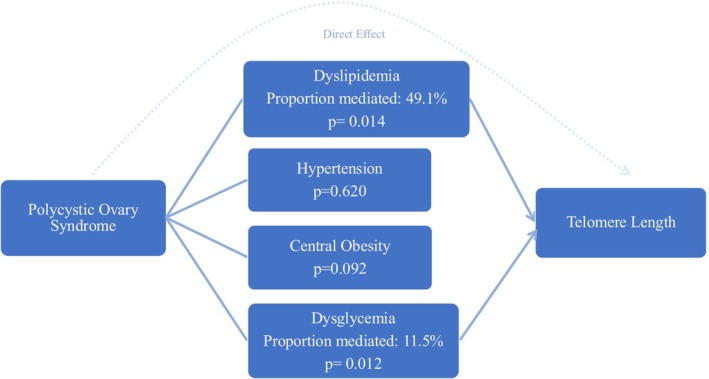
Mediation analysis showing dysglycemia and dyslipidemia partially mediated the association between polycystic ovary syndrome (PCOS) and relative leukocyte telomere length (rLTL). Data can be referred to Table S9.

While PCOS is known to be associated with cardiometabolic risk factors, such as obesity, insulin resistance and dyslipidemia, all of which were shown to be correlated with shorter rLTL, the negative relation between PCOS and rLTL appeared to be independent [12, 34, 35]. Nonetheless, the overall effects can be partially mediated. To further illustrate the direct effects of PCOS and to evaluate for the possible causal mediation effects by the cardiometabolic traits, mediation analysis was performed as shown in Table S9 with illustration as Figure 1. In all four models assessing the mediating effects of central obesity, dyslipidemia, dysglycemia and hypertension, the average direct effects of PCOS remained significant (all *p* < 0.05). Additionally, PCOS is associated with shorter rLTL through the effects of dyslipidemia and dysglycemia, but not central obesity or hypertension. The estimated proportion of effects mediated by dyslipidemia and dysglycemia were 49.1% [CI: 9.6%–99.8%; *p* = 0.014] and 11.5% [CI: 2.2%–26.7%; *p* = 0.012] respectively.

### 3.3 The Association of rLTL and Cardiometabolic Traits/Sex Hormones in PCOS Subjects

We further assessed the correlation between rLTL and various metabolic traits/sex hormones amongst women with PCOS (Table 3). An inverse association was identified between rLTL and LDL using simple linear regression (*β* = −0.235; *p* = 0.006), while longer rLTL was associated with increased HDL (*β* = 0.105; *p* = 0.048). TG, FBG and HOMA IR were not correlated. The association between rLTL and LDL remained significant after adjusting for age, but was rendered non‐significant after further adjusting for waist circumference. The association between rLTL and HDL was lost after adjusting for age. Testosterone, androstenedione and LH/FSH ratio, the hallmark hormonal changes in PCOS, were not associated with rLTL after adjustment, although higher level of testosterone and androstenedione appear to be linked to longer rLTL in univariate analyses. A summary of the linear regression models is presented in Table 3.

**TABLE 3:** Association of rLTL and cardiometabolic traits/sex hormones in PCOS patients during follow‐up using multiple linear regression.

	Model 1	Model 2	Model 3	Model 4
Triglyceride	−0.088 (−0.302 to 0.126)	−0.042 (−0.260 to 0.175)	0.027 (−0.179 to 0.233)	0.180 (−0.179 to 0.233)
	*R* ^2^ = −0.002	*R* ^2^ = 0.013	*R* ^2^ = 0.138	*R* ^2^ = 0.139
	*p* = 0.420	*p* = 0.701	*p* = 0.799	*p* = 0.865
LDL	−0.235 (−0.401 to −0.069)	−0.209 (−0.378 to −0.040)	−0.165 (−0.179 to 0.233)	−0.157 (−0.333 to 0.003)
	*R* ^2^=0.033	*R* ^2^ = 0.039	*R* ^2^ = 0.061	*R* ^2^ = 0.062
	*p* = 0.006	*p* = 0.016	*p* = 0.054	*p* = 0.068
HDL	0.105 (0.001 to 0.209)	0.094 (−0.013 to 0.200)	0.044 (−0.052 to 0.140)	0.035 (−0.052 to 0.140)
	*R* ^2^ = 0.014	*R* ^2^ = 0.015	*R* ^2^ = 0.202	*R* ^2^ = 0.209
	*p* = 0.048	*p* = 0.084	*p* = 0.369	*p* = 0.475
FBG	−0.311 (−0.739 to 0.118)	−0.177 (−0.606 to 0.253)	−0.101 (−0.483 to 0.280)	−0.071 (−0.483 to 0.280)
	*R* ^2^ = 0.005	*R* ^2^ = 0.042	*R* ^2^ = 0.226	*R* ^2^ = 0.232
	*p* = 0.154	*p* = 0.419	*p* = 0.601	*p* = 0.718
HOMA IR	−0.328 (−1.12 to 0.474)	−0.414 (−1.23 to 0.253)	−0.041 (−0.743 to 0.661)	−0.026 (−0.743 to 0.661)
	*R* ^2^ = −0.002	*R* ^2^ = −0.002	*R* ^2^ = 0.277	*R* ^2^ = 0.273
	*p* = 0.421	*p* = 0.321	*p* = 0.908	*p* = 0.942
Testosterone	0.141 (0.003 to 0.279)	0.065 (−0.087 to 0.216)	0.089 (−0.064 to 0.241)	N/A
	*R* ^2^ = 0.015	*R* ^2^ = 0.036	*R* ^2^ = 0.050	
	*p* = 0.046	*p* = 0.402	*p* = 0.252	
Androstenedione	0.045 (0.005 to 0.085)	0.017 (−0.031 to 0.065)	0.021 (−0.027 to 0.068)	N/A
	*R* ^2^ = 0.019	*R* ^2^ = 0.034	*R* ^2^ = 0.047	
	*p* = 0.028	*p* = 0.497	*p* = 0.400	
LH: FSH ratio	0.037 (−0.044 to 0.604)	0.010 (−0.073 to 0.091)	0.021 (−0.061 to 0.103)	N/A
	*R* ^2^ = −0.001	*R* ^2^ = 0.034	*R* ^2^ = 0.046	
	*p* = 0.367	*p* = 0.825	*p* = 0.611	

*Note:* Model 1: Unadjusted odds ratios. Model 2: Adjusted for age. Model 3: Adjusted for age and waist circumference. Model 4: Adjusted for age, waist circumference and testosterone.

### 3.4 rLTL Is Associated With Central Obesity, Dyslipidemia and Dysglycemia in Women With PCOS

While the above analyses implicated the potential relations between rLTL and cardiometabolic traits amongst PCOS subjects, the association appeared to be weak. Whether or not the association will be significant only when the cardiometabolic traits deviate more from the normal range remained unclear. We hence assessed the possible links between rLTL and cardiometabolic outcomes in women with PCOS, using logistic regression (Table 4). rLTL was negatively associated with dysglycemia and dyslipidemia (both *p* < 0.05) on univariable analyses, but not central obesity or hypertension. Three multiple logistic regression models were then constructed (model 2–4) to evaluate the independent association between rLTL and these outcomes. After adjusting for age, waist circumference and DM, rLTL was significantly correlated with dyslipidemia (OR: 0.563; 95% CI: 0.317–0.968; *p* = 0.042). The association between rLTL and dysglycemia was no longer significant after adjusting for age. We also defined short rLTL as rLTL less than the mean rLTL of controls to assess the correlation of shortened rLTL with central obesity, dysglycemia, dyslipidemia and hypertension (Table 4). Interestingly, shortened rLTL was associated with increased odds of dysglycemia, after adjusting for age, waist circumference and family history of DM (OR: 2.09; 95% CI: 1.04–4.29; *p* = 0.040). It was also correlated with an increased odds of central obesity after adjusting for age (OR: 1.82; 95% CI: 1.01–3.30; *p* = 0.047). The association between shortened rLTL and dyslipidemia was significant after adjusting for age, but lost after further adjusting for waist circumference.

**TABLE 4:** Association of rLTL/shortened rLTL and cardiometabolic outcomes in PCOS patients using logistic regression.

	Model 1	Model 2	Model 3	Model 4
rLTL				
Central obesity	0.715 (0.457–1.10)	0.768 (0.487–1.20)	N/A	N/A
	*p* = 0.133	*p* = 0.247		
Dyslipidemia	0.469 (0.281–0.756)	0.533 (0.315–0.869)	0.592 (0.336–1.01)	0.563 (0.317–0.968)
	*p* = 0.003	*p* = 0.014	*p* = 0.061	*p* = 0.042
Dysglycemia	0.616 (0.392–0.952)	0.705 (0.443–1.11)	0.768 (0.459–1.27)	0.758 (0.450–1.26)
	*p* = 0.032	*p* = 0.133	*p* = 0.305	*p* = 0.289
Hypertension	0.996 (0.636–1.57)	1.18 (0.740–1.92)	1.47 (0.870–2.55)	1.51 (0.878–2.66)
	*p* = 0.142	*p* = 0.154	*p* = 0.484	*p* = 0.986
Shortened rLTL				
Central obesity	1.94 (1.08–3.49)	1.82 (1.01–3.30)	N/A	N/A
	*p* = 0.027	*p* = 0.047		
Dyslipidemia	2.19 (1.21–4.00)	1.97 (1.07–3.65)	1.50 (0.762–2.93)	1.55 (0.772–3.06)
	*p* = 0.010	*p* = 0.030	*p* = 0.239	*p* = 0.201
Dysglycemia	2.66 (1.44–5.06)	2.39 (1.27–4.62)	2.07 (1.04–4.25)	2.09 (1.04–4.29)
	*p* = 0.002	*p* = 0.007	*p* = 0.042	*p* = 0.040
Hypertension	0.979 (0.534–1.817)	0.827 (0.438–1.57)	0.538 (0.254–1.12)	0.505 (0.237–1.06)
	*p* = 0.946	*p* = 0.559	*p* = 0.099	*p* = 0.072

*Note:* Model 1: Unadjusted odds ratios. Model 2: Adjusted for age. Model 3: Adjusted for age and waist circumference. Model 4: Adjusted for age, waist circumference and DM/family history of DM.

### 3.5 The Causal Effect of Telomere Length on Polycystic Ovary Syndrome

In order to further investigate whether there are causal links between shortened rLTL and PCOS, we conducted bidirectional MR analyses to examine the causal relationship between rLTL and PCOS. Given that larger sample sizes and a larger number of identified SNPs would help to robustness of MR analyses, we chose to undertake the MR analyses using data from the European population, where there is more available genetic data. A total of 153 SNPs significantly associated with rLTL were obtained (Table S1). The SNPs explained 3.57% of the variance for rLTL, and the corresponding F‐statistic was 114.37. In MR analysis, the standard IVW method indicated no causal effects of rLTL on PCOS (OR = 0.861, 95% CI 0.650–1.142, *p* = 0.299) (Table S3; Figure 1). We also applied sensitivity analyses including maximum likelihood, weighted median, weighted mode and MR‐Egger methods, which yielded similar results (Table S3; Figure 1). Heterogeneity tests indicated no heterogeneity of the genetic variants for PCOS (*p* = 0.182) (Table S4), and pleiotropy tests indicated no pleiotropy of the genetic variants (*p* = 0.676) (Table S4). Leave‐one‐out analysis indicated that the results were still well‐powered and stable even if any single SNP is excluded (Figure S1). Altogether, our results indicated no causal effects of rLTL on PCOS by MR analysis.

### 3.6 The Causal Effects of Polycystic Ovary Syndrome on Telomere Length

To further explore the reverse association between rLTL and PCOS, we further performed bidirectional MR analysis to estimate the causal effects of PCOS on rLTL. Specifically, 14 SNPs significantly associated with PCOS were obtained, respectively (Table S2). By using the IVW method, our results indicated no causal effects of PCOS (*β* = −6.75E‐04, 95% CI −0.015 to 1.014, *p* = 0.927) on rLTL (Table S5). Sensitivity analyses returned consistent results (Table S5; Figure 1). No heterogeneity was identified in the analysis (*p* = 0.141) (Table 3). The pleiotropy test indicated no pleiotropy (*p* = 0.317) amongst the genetic variants (Table S6). Leave‐one‐out analysis indicated that the results of our analysis had sufficient power (Figure S2). Taken together, our results indicated no causal effects of PCOS on rLTL by MR analysis.

## 4 Discussion

In the present study, we used an updated and refined real‐time quantitative PCR assay to evaluate the association between rLTL and PCOS, as well as that between rLTL and metabolic traits/outcomes. Our main findings were: (1) rLTL was independently and inversely associated with PCOS in Chinese subjects after adjusting for age, BMI, smoking, HOMA IR and LDL. (2) The shorter rLTL in PCOS patients is partially mediated through dyslipidemia and dysglycemia, while a direct effect of PCOS is also present. (3) Shorter rLTL was associated with cardiometabolic traits amongst PCOS patients. (4) Bi‐directional Mendelian Randomization analysis suggest no causal relations between genetically determined PCOS and telomere length.

### 4.1 Women With PCOS Have Shorter rLTL Compared With Control

To date, there have been several studies comparing the telomere length in women with and without PCOS. In line with our data, Li et al. reported shorter rLTL in PCOS subjects in their study consisting of 698 PCOS subjects and 611 healthy controls, which is the largest cohort thus far [19]. Meanwhile, contradictory findings were produced from two similar but smaller studies. Pedroso et al. reported a cohort of 150 PCOS patients and 124 healthy controls, and they found no significant difference in LTL [21]. Another study from Argentina, including 170 PCOS patients and 64 controls, reported longer telomere length in the PCOS group [36]. These discrepancies highlighted the complexity of telomere biology and could potentially represent the effects of various factors including differences in clinical characteristics, methodological variability in LTL measurements as well as the phenotypic heterogeneity of PCOS.

While real‐time quantitative PCR‐based assay offers a relatively simple and high‐throughput workflow for rLTL measurements, issues around variability and reproducibility have been a concern. Although overall the assays are similar between groups, subtle differences, such as the reference single copy gene, could have substantial effects on the results. For example, the single‐copy gene 36B4/RPLP0 used in the work by Pedroso et al. is known to have multiple processed pseudogenes, which makes it unsuitable as a reference gene [37]. In the present study, we employed an optimised protocol which has been previously shown to have low intra‐plate and inter‐plate CVs with high reproducibility [23]. In addition, both the present study and that by Li et al. consisted of Chinese subjects, while the other two studies recruited participants from the South America. Racial and ethnic differences have been shown to be associated with different clinical manifestations, phenotypes, sex hormone profile and cardiometabolic risks in women with PCOS, which could potentially influence rLTL [38, 39, 40]. Notably, Asian patients with PCOS have been reported to have milder hyperandrogenic phenotypes but higher cardiometabolic risk, both of which could be associated with shorter rLTL [12, 36, 41]. Third, the discrepancy in androgen levels across various studies may partly explain the differences as testosterone may be associated with longer LTL in PCOS patients, as reported in one of the previous studies and by the simple linear regression in our cohort, as discussed in the next section [36]. In our study, the mean testosterone level or percentage of patients with hyperandrogenism was significantly lower than in the abovementioned studies (1.40 nmol/L vs. 3.12 nmol/L) (58.2% vs. 84.2%). These factors may have contributed to our study detecting a clear difference in rLTL and an inverse relation between rLTL and PCOS using logistic regression.

### 4.2 Cardiometabolic Traits Partially, but Not Fully, Mediate the Effects of PCOS on rLTL

Notably, previous literature suggested an inverse association between telomere length and numerous cardiometabolic traits including BMI, WHR, LDL, FBG, HOMA‐IR, SBP and DBP in the general population, while PCOS patients are known to be at risk of these metabolic derangement [12].

To our knowledge, although the aforementioned studies aimed to compare the telomere length amongst PCOS and control subjects, we are the first group to investigate both the independent association and the potential indirect effects of PCOS on rLTL through known cardiometabolic confounders of shortened rLTL. In our study, we were able to, for the first time, demonstrated that rLTL remained independently inversely associated with PCOS after adjusting for various cardiometabolic traits, in contrast with the previous study which only adjusted for age [19]. We also further dissected the potential causal‐mediation relations between PCOS, cardiometabolic outcomes and rLTL by mediation analysis. We were able to show that dysglycemia and dyslipidemia partially contributed to the shortened rLTL in PCOS patients, while there remained a direct effect of PCOS on rLTL that could not be accounted by the metabolic derangements. This implicates the presence of other pathophysiological mechanisms underlying the difference in rLTL.

In women with PCOS, we found that androgen levels, including testosterone and androstenedione, were positively correlated with rLTL in simple linear models (Testosterone: *β* = 0.141; *p* = 0.046; Androstenedione: *β* = 0.536; *p* = 0.028), suggesting the potential effects of androgens on telomere length. Interestingly, the associations as shown with our data became non‐significant after adjusting for age. Whether the named association represents a true biological relation, or it only reflects the higher androgen levels in younger women remained unclear and warranted further investigations. Owing to the lack of data on sex hormone levels in the control subjects, we have not been able to compare both groups and to further evaluate the effects of hormonal disturbance.

Hence, how PCOS is related to shortened rLTL beyond metabolic derangement require further efforts, and could potentially be due to hormonal disturbance, chronic inflammation, increased oxidative stress and other mechanisms in PCOS subjects [42]. Of note, increased circulating inflammatory markers such as homocysteine, interleukin‐6, tumour necrosis factor α and so forth, have been shown in women with PCOS [42]. Markers of oxidative stress, such as reduced GSH/GSSH ratio, increased level of reactive oxygen species from mononuclear cells, and upregulated superoxide dismutase activity have also been demonstrated [43, 44]. Whether these represent the underlying mechanisms of shortening of rLTL in PCOS subjects remains unclear.

### 4.3 Association of Metabolic Traits and rLTL in PCOS Subjects

Adding to earlier studies, our group is also the first to evaluate the relationship between rLTL and a comprehensive panel of cardiometabolic traits in PCOS subjects. In addition to Li et al.'s study, which explored the relations between LTL and glycemic indices, we further investigated the association between rLTL, adiposity, lipid profile and blood pressure in PCOS subjects. Similar to their analyses, no significant relationship was found between glycemic indices and rLTL, but we reported that rLTL was positively associated with HDL and inversely associated with LDL in univariable analyses. The loss of association after adjusting for waist circumference may suggest the critical role of visceral adiposity in lipid derangement, as well as shortened rLTL in PCOS patients. There is strong evidence that aberrant adipocyte function in PCOS women could lead to insulin resistance, dyslipidemia and subclinical inflammation [45, 46, 47]. Visceral adiposity has been reported to be associated with shorter LTL as well [12].

Furthermore, when dyslipidemia was defined as a composite outcome representing derangement in one or more lipid traits, namely HDL, LDL and/or TG, the association becomes more robust. It remained statistically significant after adjusting for confounders including age, waist circumference and DM. Dysglycemia, also defined with both fasting glucose and/or OGTT, also correlated with shortened rLTL in PCOS patients after adjusting for age, waist circumference and family history of DM. These results further supported that rLTL is closely related to cardiometabolic traits in PCOS patients, and could serve as a potential biomarker reflecting the burden of these traits.

### 4.4 No Direct Causal Relation Between PCOS and rLTL

Given the significantly shorter rLTL in PCOS subjects, we performed a bi‐directional Mendelian Randomization analysis to assess whether there is a causal links between rLTL and PCOS, or vice versa. The negative results suggested no evidence of causal relations and again, the shortened rLTL in PCOS is likely to be mediated by other factors such as metabolic changes, hormonal disturbance and pro‐inflammatory cytokines in PCOS [42, 46]. Further studies may be needed to elucidate the mechanism by which rLTL are shortened in PCOS patients.

### 4.5 Limitation

The present study provided evidence that TL attrition may be associated with PCOS and its cardiometabolic consequences. However, it is not without limitation. First, this study is not powered to detect relatively weak association given the limited sample size. Second, although the optimised PCR‐based assay may contribute to higher reproducibility, it only reflects the relative average LTL, instead of providing a detailed profiling of TL distribution across different chromosomes and cells. Third, the potential relationship between rLTL and sex hormone level cannot be evaluated due to the lack of sex hormone measurement in the control group.

## 5 Conclusions

Chinese women with PCOS have significantly shortened rLTL compared to controls. rLTL is inversely associated with the risk of PCOS, independently of metabolic traits. Shortened rLTL is associated with an increased risk of dyslipidemia and dysglycemia in women with PCOS.

## Author Contributions

R.N.C.C. measured telomere length, performed statistical analysis, and wrote the draft manuscript. F.C., K.K.W., H.C.H.T., A.C.W.N. and C.K.P.L. contributed to study logistics and the telomere experiments. C.H. conducted the MR analysis. M.S. and J.C.N.C. contributed to the sequencing studies and calculation of absolute telomere length. N.Y.H.N., A.Y.T.T., L.P.C., W.H.T. and R.C.W.M recruited subjects and conducted evaluation. C.H.T.T assisted with data analysis. C.C.W. assisted with data interpretation. M.V.J., A.J.J., and A.A.H. developed and refined the modified LTL measurement method. C.K.P.L. and R.C.W.M. designed the research, obtained funding support, supervised the research work, and wrote the manuscript. All authors contributed to the writing of the manuscript and approved the final version. R.C.W.M. is the guarantor of this work and, as such, had full access to all the data in the study and takes responsibility for the integrity of the data and the accuracy of the data analysis.

## Conflicts of Interest

J.C.N.C. received consultancy fees from Astra Zeneca, Bayer, Boehringer Ingelheim, Celltrion, MSD, Pfizer, Servier and Viatris Pharmaceutical, speaker fees from Astra Zeneca, Bayer, Boehringer Ingelheim, MSD, Merck, Sanofi and Servier and research grants through her institutions from Applied Therapeutics, Astra Zeneca, Hua Medicine, Lee Powder, Lilly, Merck and Servier. R.C.W.M. has received research grants for clinical trials from AstraZeneca, Bayer, MSD, Novo Nordisk, Sanofi, Tricida Inc. and honoraria for consultancy or lectures from AstraZeneca, Bayer, Boehringer Ingelheim, Merck and Roche Diagnostics, all used to support diabetes research at the Chinese University of Hong Kong. J.C.N.C., C.K.P.L. and R.C.W.M. are co‐founders of GemVCare, a technology start‐up initiated with support from the Hong Kong Government Innovation and Technology Commission and its Technology Start‐up Support Scheme for Universities (TSSSU). A.J.J. has received research grants for clinical trials from Abbott and Sanofi‐Aventis, and honoraria for consultancy for Abbott, Amgen, Medtronic and Sanofi‐Aventis. No other potential conflicts of interest relevant to this article were reported.

## Supporting information


**Figure S1.** Odd ratios of PCOS per unit increase in genetically proxied telomere length.

**Figure S2.** Effect size of genetically determined PCOS on telomere length.

Data S1.

Data S2.

## Data Availability

Some or all datasets generated during and/or analysed during the current study are not publicly available but are available from the corresponding author on reasonable request.
