# Supplementary material for: Shortened Relative Leukocyte Telomere Length Is Associated With Polycystic Ovary Syndrome and Metabolic Traits
Source: Endocrinol Diabetes Metab. 2025 Feb 18;8(2):e70030. doi: 10.1002/edm2.70030 (PMC11833164; doi:10.1002/edm2.70030)
Supplement: Supplementary file 1 — Figure S1. Odd ratios of PCOS per unit increase in genetically proxied telomere length. [file EDM2-8-e70030-s004.pdf]

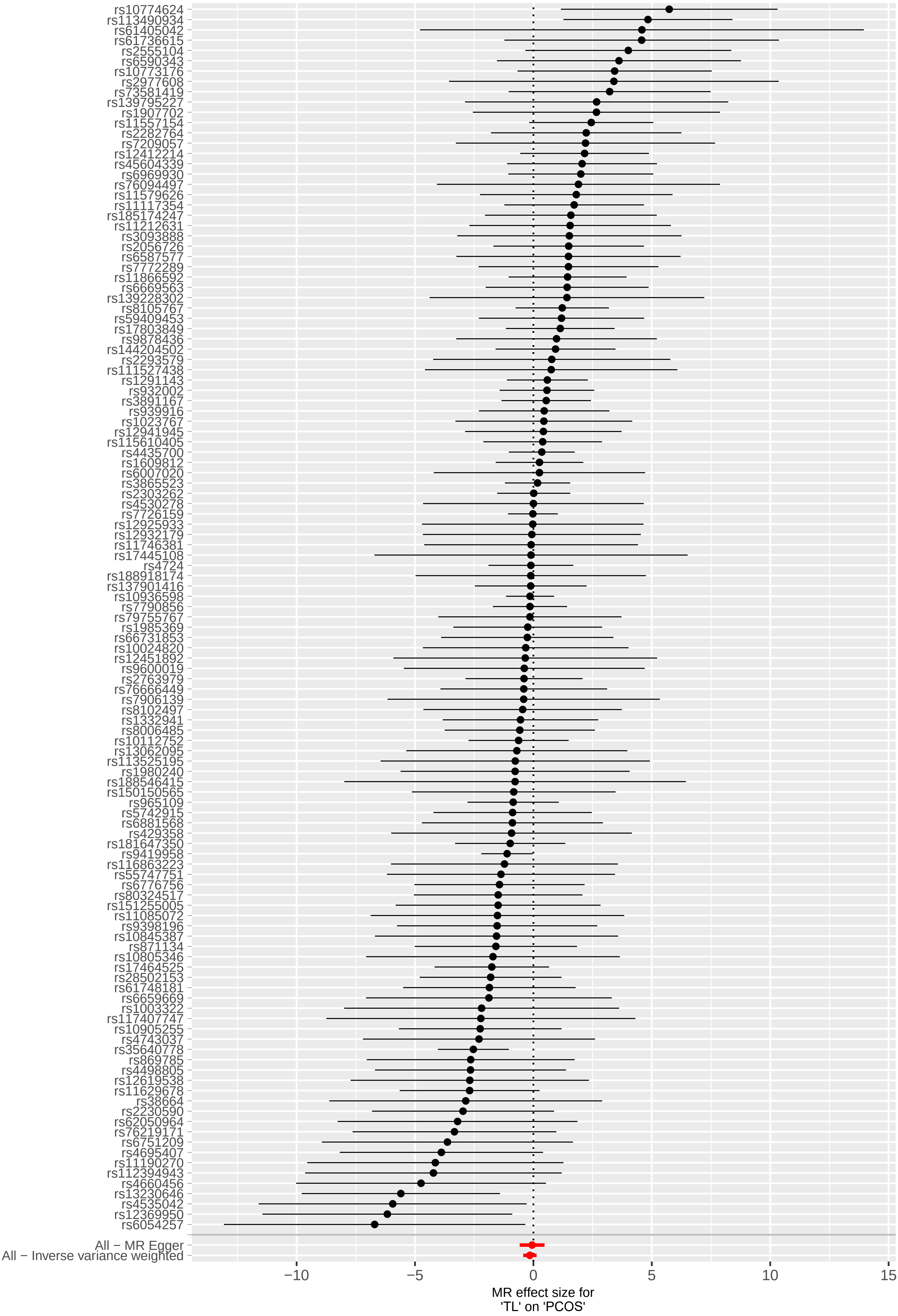
rs10774624
rs113490934
rs61405042
rs61736615
rs2555104
rs6590343
rs10773176
rs2977608
rs73581419
rs139795227
rs1907702
rs11557154
rs2282764
rs7209057
rs12412214
rs45604339
rs6969930
rs76094497
rs11579626
rs11117354
rs185174247
rs11212631
rs3093888
rs2056726
rs6587577
rs7772289
rs11866592
rs6669563
rs139228302
rs8105767
rs59409453
rs17803849
rs9878436
rs144204502
rs2293579
rs111527438
rs1291143
rs932002
rs3891167
rs939916
rs1023767
rs12941945
rs115610405
rs4435700
rs1609812
rs6007020
rs3865523
rs2303262
rs4530278
rs7726159
rs12925933
rs12932179
rs11746381
rs17445108
rs4724
rs188918174
rs137901416
rs10936598
rs7790856
rs79755767
rs1985369
rs66731853
rs10024820
rs12451892
rs9600019
rs2763979
rs76666449
rs7906139
rs8102497
rs1332941
rs8006485
rs10112752
rs13062095
rs113525195
rs1980240
rs188546415
rs150150565
rs965109
rs5742915
rs6881568
rs429358
rs181647350
rs9419958
rs116863223
rs55747751
rs6776756
rs80324517
rs151255005
rs11085072
rs9398196
rs10845387
rs871134
rs10805346
rs17464525
rs28502153
rs61748181
rs6659669
rs1003322
rs117407747
rs10905255
rs4743037
rs35640778
rs869785
rs4498805
rs12619538
rs11629678
rs38664
rs2230590
rs62050964
rs76219171
rs6751209
rs4695407
rs11190270
rs112394943
rs4660456
rs13230646
rs4535042
rs12369950
rs6054257
All − MR Egger
All − Inverse variance weighted
−10
−5
0
5
10
15
MR effect size for
'TL' on 'PCOS'
