# Supplementary figures and images for: Shortened Relative Leukocyte Telomere Length Is Associated With Polycystic Ovary Syndrome and Metabolic Traits
Source: Endocrinol Diabetes Metab. 2025 Feb 18;8(2):e70030. doi: 10.1002/edm2.70030 (PMC11833164; doi:10.1002/edm2.70030)

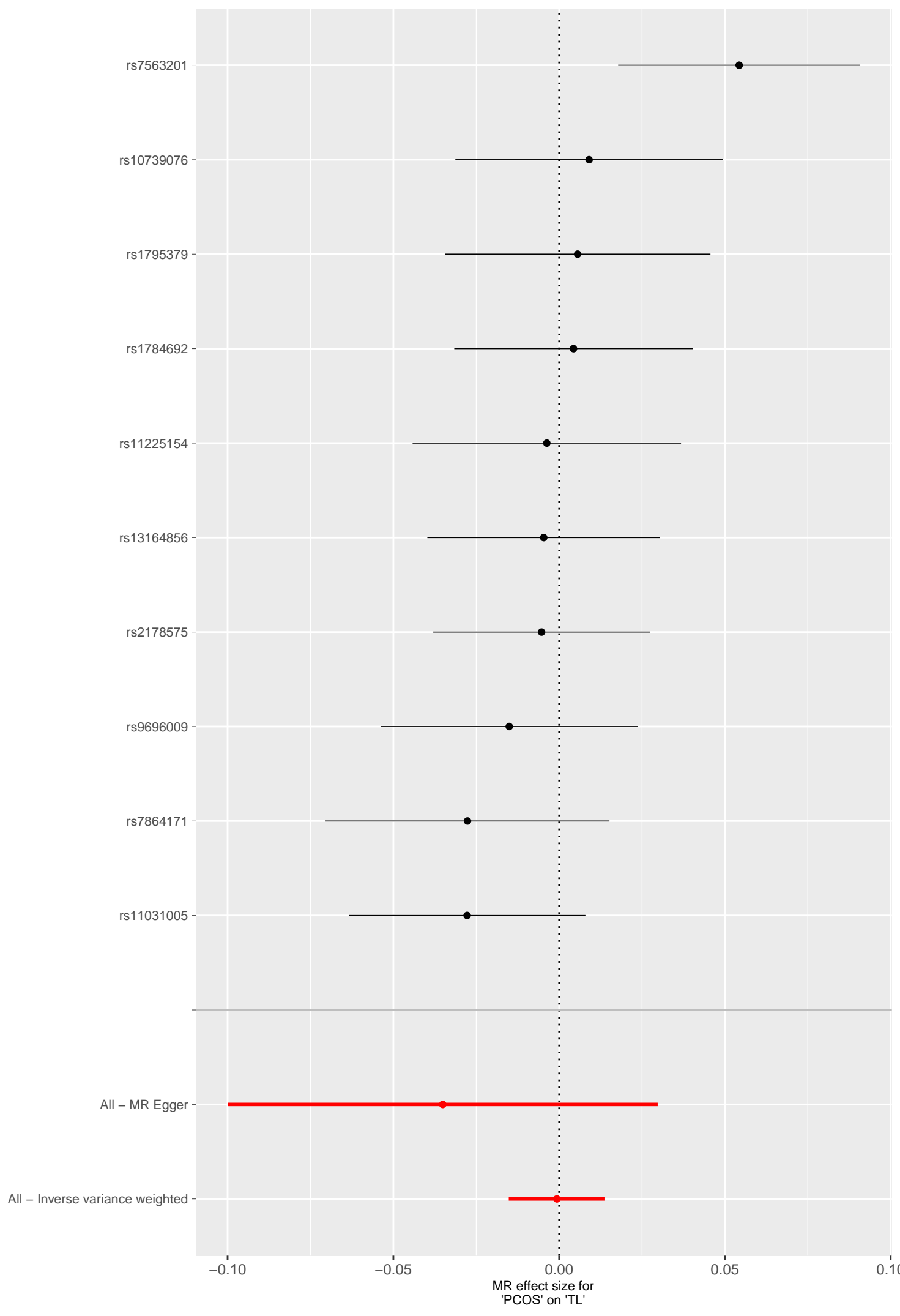

Supplement: Supplementary file 2 — Figure S2. Effect size of genetically determined PCOS on telomere length. [file EDM2-8-e70030-s003.pdf]
