# Supplementary material for: Shortened Relative Leukocyte Telomere Length Is Associated With Polycystic Ovary Syndrome and Metabolic Traits
Source: Endocrinol Diabetes Metab. 2025 Feb 18;8(2):e70030. doi: 10.1002/edm2.70030 (PMC11833164; doi:10.1002/edm2.70030)
Supplement: Supplementary file 4 — Data S2. [file EDM2-8-e70030-s002.docx]

|  | Odds Ratio | 95% CI | p-Value |
| --- | --- | --- | --- |
| Model 1 | 0.900 | 0.857-0.944 | <0.001 |
| Model 2 | 0.906 | 0.862-0.951 | <0.001 |
| Model 3 | 0.909 | 0.864-0.954 | <0.001 |
| Model 4 | 0.908 | 0.863-0.954 | <0.001 |
| Model 5 | 0.913 | 0.864-0.962 | <0.001 |
| Model 6 | 0.906 | 0.857-0.956 | <0.001 |

Supplementary Table 7: Association of estimated absolute LTL and PCOS Status using Simple and Multivariable Logistic Regression

Model 1: Unadjusted odds ratio. Model 2: Adjusted for age; Model 3: Adjusted for age and waist circumference; Model 4: Adjusted for age, waist circumference, and smoking; Model 5: Adjusted for age, waist circumference, smoking and HOMA IR. Model 6: Adjusted for age, waist circumference, smoking, HOMA IR and LDL.

|  | Odds Ratio | 95% CI | p-Value |
| --- | --- | --- | --- |
| Model 1 | 0.482 | 0.342-0.668 | <0.001 |
| Model 2 | 0.505 | 0.358-0.703 | <0.001 |
| Model 3 | 0.519 | 0.360-0.738 | <0.001 |
| Model 4 | 0.517 | 0.359-0.736 | <0.001 |
| Model 5 | 0.533 | 0.366-0.766 | <0.001 |
| Model 6 | 0.496 | 0.336-0.721 | <0.001 |

Supplementary Table 8: Sensitivity Analyses for Association of rLTL and PCOS Status using Simple and Multivariable Logistic Regression

Model 1: Unadjusted odds ratio. Model 2: Adjusted for age; Model 3: Adjusted for age and BMI; Model 4: Adjusted for age, BMI, and smoking; Model 5: Adjusted for age, BMI, smoking and fasting glucose. Model 6: Adjusted for age, BMI, smoking, fasting glucose and LDL.

|  | Estimate | 95% CI | p-Value |
| --- | --- | --- | --- |
| Central Obesity | | | |
| Average Causal Mediation Effects | -0.016 | -0.040 to 0.002 | 0.092 |
| Average Direct Effects | -0.260 | -0.374 to -0.145 | <0.001 |
| Total Effects | -0.276 | -0.386 to -0.166 | <0.001 |
| Proportion Mediated | 0.059 | -0.007 to 0.172 | 0.092 |
|  | | | |
| Dyslipidemia | | | |
| Average Causal Mediation Effects | -0.135 | -0.233 to -0.024 | 0.014 |
| Average Direct Effects | -0.141 | -0.290 to -0.001 | 0.049 |
| Total Effects | -0.276 | -0.386 to -0.166 | <0.001 |
| Proportion Mediated | 0.491 | 0.096-0.998 | 0.014 |
|  | | | |
| Dysglycemia |  |  |  |
| Average Causal Mediation Effects | -0.032 | -0.005 to -0.007 | 0.012 |
| Average Direct Effects | -0.244 | -0.374 to -0.122 | <0.001 |
| Total Effects | -0.276 | -0.401 to -0.153 | <0.001 |
| Proportion Mediated | 0.115 | 0.022-0.267 | 0.012 |
|  | | | |
| Hypertension | | | |
| Average Causal Mediation Effects | -0.015 | -0.074 to 0.047 | 0.620 |
| Average Direct Effects | -0.261 | -0.386 to -0.139 | <0.001 |
| Total Effects | -0.276 | -0.386 to -0.166 | <0.001 |
| Proportion Mediated | 0.056 | -0.174 to 0.276 | 0.620 |

Supplementary Table 9: Mediation Analysis of PCOS on rLTL through Cardiometabolic Risk Factors.
